# Recombinant p40 Protein Promotes Expression of Occludin in HaCaT Keratinocytes: A Brief Communication

**DOI:** 10.3390/microorganisms11122913

**Published:** 2023-12-03

**Authors:** Carolina Domínguez-Díaz, Karina Elizabeth Avila-Arrezola, Jorge A. Rodríguez, Susana del-Toro-Arreola, Vidal Delgado-Rizo, Mary Fafutis-Morris

**Affiliations:** 1 Doctoral Program in Biomedical Sciences, Physiology Department, Centro Universitario de Ciencias de la Salud, Universidad de Guadalajara, Guadalajara 44340, Mexico; carolina.dominguezd@alumnos.udg.mx; 2 Immunology and Dermatology Research Center (CIINDE), Zapopan 45190, Mexico; karyelinuta@gmail.com; 3 Department of Industrial Biotechnology, Centro de Investigación y Asistencia en Tecnología y Diseño del Estado de Jalisco, Zapopan 45019, Mexico; jrodriguez@ciatej.mx; 4 Physiology Department, Centro Universitario de Ciencias de la Salud, Universidad de Guadalajara, Guadalajara 44340, Mexico; susana@cucs.udg.mx (S.d.-T.-A.); vidalrizo@gmail.com (V.D.-R.)

**Keywords:** postbiotic, epithelial barriers, epithelial cells, tight junctions, p40

## Abstract

The ability of epithelial barriers to perform as the first defense line against external damage derives from tight junctions, protein complexes that block microorganisms through the paracellular space. Indeed, disturbances of barrier permeability caused by bacterial metabolites and other inflammatory stimuli are the consequence of changes in protein expression in these complexes. Postbiotics, molecules derived from bacteria with beneficial effects on the host, improve barrier function through the activation of survival pathways in epithelial cells. *Lacticaseibacillus rhamnosus* GG secretes the muramidase p40, which protects intestinal barriers through an EGFR-dependent pathway. In this work, we cloned, expressed, and purified the recombinant p40 protein from *L. rhamnosus* GR-1 to evaluate its effect on cell viability, cell cytotoxicity, TEER, and protein levels of tight junctions, as well as EGFR activation via Western blot on HaCaT keratinocytes subjected to LPS. We found a novel mutation at residue 368 that does not change the structure of p40. Our protein also reduces the LPS-induced increase in cell cytotoxicity when it is added prior to this stimulus. Furthermore, although LPS did not cause changes in barrier function, p40 increased TEER and occludin expression in HaCaT, but unlike previous work with p40 from LGG, we found that recombinant p40 did not activate EGFR. This suggests that recombinant p40 enhances epithelial barrier function through distinct signaling pathways.

## 1. Introduction

The human body possesses physical barriers made of tightly joined cells to limit the access of surrounding pathological and harmful agents. These barriers are called epithelial barriers, tissues that cover the body's surfaces in contact with the external environment, such as the skin and the respiratory, genitourinary, and gastrointestinal tracts. Their integrity, which allows for the homeostasis of our system, is the consequence of the expression of specific protein complexes that regulate the passage of molecules through these barriers. For this reason, these protein complexes are called tight junctions.

Tight junctions are one of the different types of intercellular junctions between epithelial cells. They are responsible for the selective barrier function, meaning the exchange of molecules specific to each organ, as well as preventing the entry of microorganisms and thus playing a key role as regulators of the inflammatory processes. Tight junctions include both transmembrane proteins, which are responsible for the paracellular barrier, and periphery proteins to form a cytosolic plaque that stabilizes and allows the assembly and disassembly of these complexes in the epithelial cell [1].

Expressing the proteins of tight junctions is important to maintain the epithelial barrier function. One way to achieve this is with molecules derived from bacteria that have beneficial properties for the health of the organism, which are known as postbiotics. According to The International Scientific Association for Probiotics and Prebiotics (ISAPP) postbiotics are defined as a “preparation of inanimate microorganisms and/or their components that confers a health benefit on the host” [2]. These molecules include cell-free supernatants, cell wall fragments, bacterial lysates, short-chain fatty acids, enzymes, exopolysaccharides, amino acids, peptides, and fermentation products [3]. They compete against pathogens; maintain the integrity of the intestinal barrier; and possess immunomodulatory, antimicrobial, and antitumoral activity without the risks associated with live microbes [3,4]. *Lacticaseibacillus rhamnosus* GG (LGG) secretes p40, a 40 kDa protein that has been widely studied as a potential postbiotic with important anti-apoptotic, anti-inflammatory, and immunomodulatory properties in both in vitro and in vivo models of the colonic intestinal barrier [5,6,7]. Additionally, the protective effects of p40 on the intestinal barriers can be applied to other epithelial tissues. Therefore, this study aims to evaluate the effects of a recombinant p40 protein (rt-p40) obtained from *L. rhamnosus* GR-1 on the cell viability, cell cytotoxicity, transepithelial electrical resistance (TEER), and expression of tight junction proteins (occludin and claudin-1) in cultures of the HaCaT epithelial cell line subjected to *E. coli* lipopolysaccharide (LPS) as an inflammatory microbiological stimulus.

## 2. Materials and Methods

### 2.1. Bacterial Growth Conditions

*L. rhamnosus* GR-1 (LMG 8153, BCCM, Ghent, Belgium) was grown in MRS medium (BD Difco^TM^, Franklin Lakes, NJ, USA) at 37 °C under static conditions. *Escherichia coli* DH10β (OriGene Technologies, Rockville, MD, USA) was used as a host for cloning. The pET28a plasmids were introduced into *E. coli* BL21 (DE3) (Sigma-Aldrich^®^, St. Louis, MO, USA) for protein expression and purification, which were grown in Luria-Bertani medium at 37 °C under agitation.

### 2.2. DNA Extraction

Genomic DNA from *L. rhamnosus* GR-1 was extracted with the Wizard^®^ Genomic DNA Purification kit (Promega Corp, Madison, WI, USA), and the protocol was followed according to the instructions of the manufacturer, with the addition of an incubation step with 10 mg/mL lysozyme at 37 °C for one hour to aid cell lysis.

### 2.3. Cloning, Expression, and Purification of p40

The primers were designed based on the genomic sequence of *L. rhamnosus* ATCC 8530. The primers P40NCOI and P40XHOI were designed to amplify the mature sequence of the p40 protein (without the signal peptide) and with additional restriction sites to facilitate the following cloning protocol (Table 1). The mature p40 sequence was amplified from the extracted genomic DNA with Q5^®^ High-Fidelity DNA polymerase (New England BioLabs^®^ Inc., Ipswich, MA, USA). The PCR conditions included initial denaturation at 98 °C for 10 s; followed by 30 cycles of 5 s for denaturation at 98 °C, 45 s for alignment at 72 °C, and 30 s for extension at 72 °C; as well as 2 min for a final extension step at 72 °C. The amplified fragments were digested with Ncol and Xhol and ligated with the T4 DNA ligase (New England BioLabs^®^ Inc.) to the pET28a(+) vector, which carries a His-tag in the C-terminal sequence for purification. The ligated product was used to transform *E. coli* DH10β, and the plasmid DNA was extracted with the SV Gel and PCR Clean-up System (Promega Corp) and PureYield™ Plasmid Miniprep System (Promega Corp) kits. The fragments were verified by DNA sequencing (Macrogen, Seoul, Republic of Korea) and then used to transform *E. coli* BL21 (DE3). Expression was induced with 0.5 mM IPTG when the bacterial density reached an OD600nm of 0.4, and growth was continued at 16 °C for 16 h. The bacterial pellets were recovered by centrifugation and resuspended in lysis buffer (20 nM phosphate buffer, 100 mM NaCl, 20 mM imidazole, at a pH of 7.2). Bacteria were lysed by sonication, and cellular debris was removed by centrifugation at 10,000 rpm at 4 °C for 10 min. The supernatant was loaded onto a HisTrap FF column (GE Healthcare Life Sciences, Chicago, IL, USA) using the GE Äkta Prime Plus FPLC system (GE Healthcare Life Sciences). The column was washed 10 times with binding buffer (20 mM phosphate buffer, 500 mM NaCl, 20 mM imidazole, at a pH of 7.2), followed by 5 washes with elution buffer (20 mM phosphate buffer, 500 mM NaCl, and 500 mM imidazole at a pH of 7.2) to remove the bound protein. Proteins were eluted using a linear gradient from 20 to 500 mM imidazole in the elution buffer. A constant flow rate of 1 mL/min was maintained throughout all purification steps. The eluted fractions were analyzed by SDS-PAGE, and the rt-p40 fractions were concentrated and dialyzed in PBS at a pH of 7.4. The concentration was determined using Bradford reagent (Sigma-Aldrich^®^) before storing at −80 °C.

### 2.4. Cell Culture and Cell Treatment

The spontaneously immortalized keratinocyte HaCaT cell line was provided by Dr. Petra Boukamp from the German Cancer Research Center (DKFZ, Heidelberg, Germany). HaCaT cells were grown in DMEM (D6429, Sigma-Aldrich^®^) medium supplemented with 10% FBS at 37 °C with an atmosphere of 5% CO_2_ and 98% humidity.

To test the effect of the rt-p40 protein on HaCaT cell viability, we seeded 1 × 10^5^ cells per well in a 96-well plate, onto which we added 10 ng/mL, 20 ng/mL, and 50 ng/mL of rt-p40 for 1 h. We performed this test with an MTT assay under these conditions.

We selected the LPS from *E. coli* O127:B8 (L3129, Sigma-Aldrich^®^) concentration from dose–response curve. We seeded 1 × 10^4^ per well in a 96-well plate, onto which we added 0.1 µg/mL, 1 µg/mL, and 10 µg/mL of LPS for 24 h to measure LDH release to assess cell death.

Before the experiments, DMEM medium with 0.5% FBS was used, and the cells were treated under different conditions: (a) cells incubated with PBS without any additional stimulus were used as a negative control, (b) 10 ng/mL rt-p40 for 1 h, (c) 1 µg/mL LPS for 24 h, (d) 10 ng/mL rt-p40 for 1 h before 1 µg/mL LPS for 24 h, and (e) 1 µg/mL LPS for 24 h before 1 h with 10 ng/mL rt-p40. The experiments were performed after incubation treatments. All experiments were performed in quadruplicate for all techniques used, except for MTT and LDH assays, which were performed in triplicate.

### 2.5. MTT Assay

We seeded 1 × 10^5^ cells per well in a 96-well plate, onto which the stimuli were added. The cells were grown in DMEM medium with 0.5% FBS and were incubated for 24 h with their corresponding stimuli. The rapid protocol of the CyQUANT™ MTT Cell Proliferation Assay Kit (Thermo Fisher Scientific, Waltham, MA, USA) was followed according to the manufacturer’s instructions. On the day of the experiment, 100 µL of PBS buffer and then 10 µL of CyQUANT™ MTT reagent were added to each well. A negative control without cells was included with only PBS + MTT. The plate was incubated for 3 h at 37 °C, after which the plate was centrifugated, 85 µL of the total volume was removed, and 50 µL of dimethyl sulfoxide was added. The plate was incubated for another 15 min, and each well was resuspended before absorbance measurement. Absorbance reading was performed with the microplate reader FlexA-200 (ALLSHENG, Hangzhou, China) at a wavelength of 540 nm.

### 2.6. LDH Assay

LDH release was measured to assess the level of cell death using the CyQUANT™ LDH Cytotoxicity Assay Kit (Thermo Fisher Scientific). We seeded 1 × 10^4^ cells per well in a 96-well plate, with the corresponding stimuli, and additional cells in triplicate wells for Spontaneous LDH activity controls and Maximum LDH activity controls to determine the minimal and maximal LDH release, respectively. After the incubation time, 10 µL water and 10 µL 10× Lysis Buffer were added for these controls, respectively, and incubated for 45 min at 37 °C. A total of 50 µL of cell supernatant from the experimental conditions and controls were added to a new 96-well plate with 50 µL Reaction Mixture and incubated at 37 °C for 30 min protected from light. To stop the reaction, 50 µL of Stop Solution was added, and the plate was incubated for an additional 1 h at room temperature. Absorbance reading was performed with the microplate reader FlexA-200 (ALLSHENG) at 490 nm and 680 nm. To determine LDH activity, the 680 nm absorbance was subtracted from the 490 nm. Percent cytotoxicity was calculated as follows: (Experimental condition − Spontaneous LDH activity)/(Maximum LDH activity − Spontaneous LDH activity) × 100.

### 2.7. TEER

Transepithelial resistance (TEER) was measured using the Millicell^®^-ERS-2 Voltohmmeter (Millipore, Burlington, MA, USA). We seeded 3 × 10^5^ cells in plates with Corning^®^ Transwell^®^ inserts, with a diameter of 6.5 mm and a pore size of 0.4 µm (CLS470, Corning Costar Corp, Corning, NY, USA). An insert without cells and with the corresponding medium served as a blank in this series of experiments. This value was subtracted from the average value obtained from our seeded inserts. Measurements were taken 7 days after the cells were seeded and stimulated.

### 2.8. Western Blot

Proteins were denatured with Laemmli buffer at 95 °C for 5 min, followed by SDS-PAGE electrophoresis to separate the proteins by molecular weight. The proteins were transferred on PVDF membranes and probed with antibodies for occludin (sc-133256, Santa Cruz Biotechnology, Inc., Dallas, TX, USA), claudin-1 (sc-166338, Santa Cruz Biotechnology, Inc.), P-EGFR (phosphorylated in Tyr 1068 h) (sc-24616, Santa Cruz Biotechnology, Inc.), total EGFR (sc-03, Santa Cruz Biotechnology, Inc.), and GAPDH (sc-47724, Santa Cruz Biotechnology, Inc.). We used m-IgGκ BP-HRP, a horseradish peroxidase-conjugated antibody (sc-516102, Santa Cruz Biotechnology, Inc.), as a secondary antibody for all primary antibodies except for total EGFR, for which the mouse anti-rabbit IgG-HRP antibody was used (sc-2357, Santa Cruz Biotechnology, Inc.). The membranes were developed via chemiluminescence (Immobilon^®^ Western Chemiluminescent HRP Substrate, WBKLS0500, Millipore) in UVP ChemStudio equipment (Analityk Jena, Jena, Germany), and densitometric analysis was performed with VisionWorks software (version 8.21, Analityk Jena).

### 2.9. Statistics

Comparison between groups was performed with 1-way ANOVA with GraphPad Prism software version 8.0.1 (GraphPad Software, Inc., La Jolla, CA, USA). Tukey’s multiple comparison tests were used. The level of statistical significance was defined at *p* < 0.05. Data are reported as mean ± SEM.

## 3. Results

### 3.1. Cloning and Purification of rt-p40 from L. rhamnosus GR-1

From the p40 gene sequencing, we performed the amino acid sequence alignment of our mature rt-p40 with the sequences from p40 from *L. rhamnosus* GR-1 and LGG, and we found a mutation at the residue 368 of the protein, which resulted in a change from a serine to an aspartic acid in rt-p40. We used BlastP to find amino acid sequences with high homology and found 97% homology with the p40 LGG sequence, despite being only different in one amino acid (Figure S1). We also aligned the mature sequences of rt-p40 and p40 from LGG with the Clustal Omega algorithm, and the characteristics of the amino acids, as well as the changes according to the physicochemical properties, are shown in Figure S2. This program classifies the change in amino acids with low similarity due to differences in charge. We used the PHYRE2 algorithm to obtain the three-dimensional structure of our rt-p40 protein [8], where we observed a coiled-coil structure at the N-terminal end and that the CHAP domain folds into β-sheet at the C-terminal end, as previously reported (Figure S3) [9,10,11]. Indeed, the results show that no changes are predicted in the structure of the protein due to the change of amino acids.

**Figure 1:**
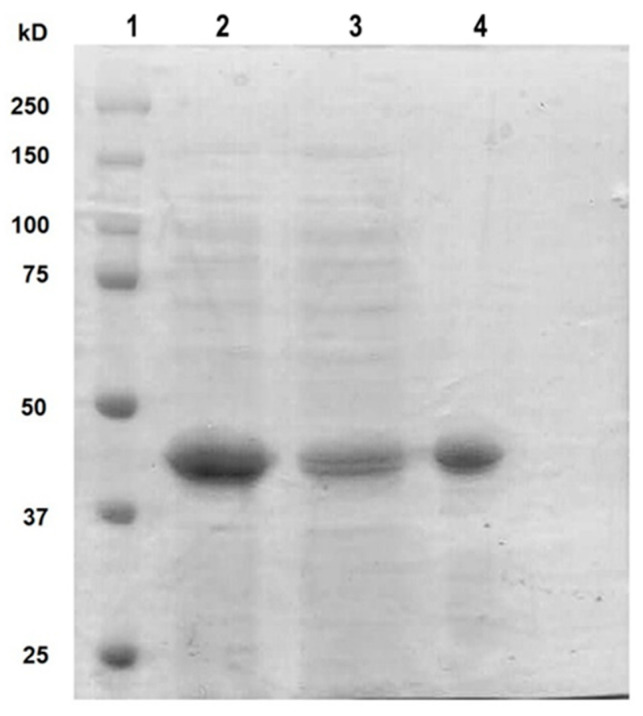
SDS PAGE with purified rt-p40. The protein ladder was loaded in lane 1. Lane 2 contains the sample before purification. In lane 3, the protein is not bound to the column. Purified rt-p40 is shown in lane 4 at the expected molecular weight.

Once the expression conditions of rt-p40 were optimized, it was purified by FPLC, where different fractions were obtained from the elution of the column. They were concentrated, dialyzed in PBS, pH 7.4, and finally filtered through a 0.22 µm membrane for their posterior use in cell culture. Figure 1 shows the SDS-PAGE gel with our original sample obtained from the lysis of *E. coli* cells in the second lane, the unbound proteins to the HisTrap FF column in the third lane, and finally, the purified rt-p40 in the fourth lane between the 37 and 50 kDa bands, as expected due to its molecular weight of 40 kDa.

### 3.2. Cell Viability and Cell Cytotoxicity

To determine the concentration of rt-p40, cell viability was evaluated with different concentrations of this protein. Our results showed that there were no statistically significant changes in cell viability under the different concentrations of rt-p40, so the concentration of 10 ng/mL of rt-p40 was selected for all subsequent experiments (Figure S4).

We also measured LDH release to assay cell cytotoxicity with different concentrations of LPS. We found that the concentrations of 1 µg/mL (*p* = 0.0336) and 10 µg/mL (*p* = 0.0067) LPS significantly increase cell cytotoxicity compared to cells without this stimulus. We also found that 10 µg/mL LPS significantly increases cell death (*p* = 0.0146) compared with 0.1 µg/mL LPS. Based on this, we selected the concentration of 1 µg/mL for all subsequent experiments (Figure S5).

**Figure 2:**
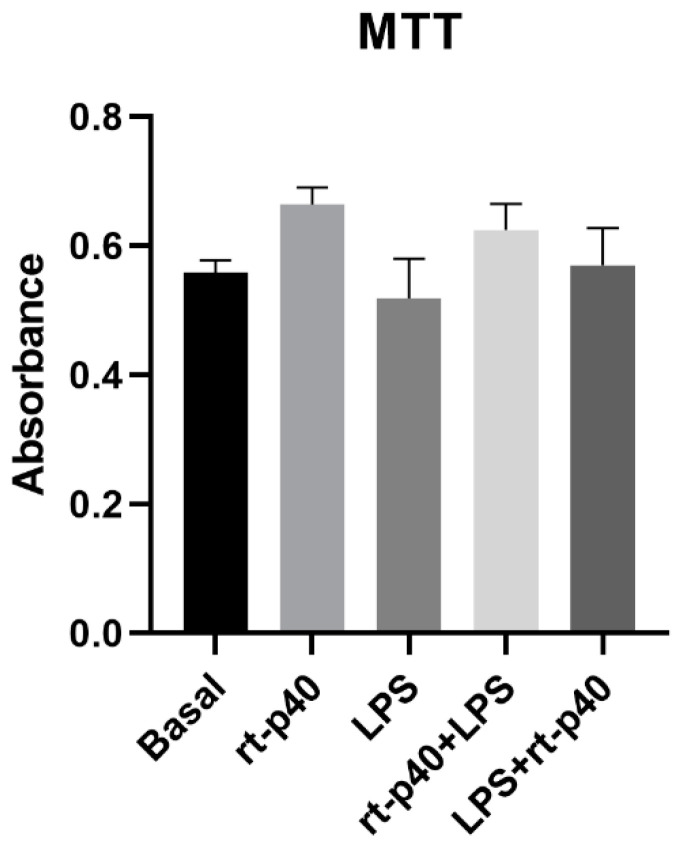
Cell viability in the HaCaT cell line under the different stimuli with rt-p40 (10 ng/mL, 1 h), LPS (1 µg/mL, 24 h), rt-p40+LPS (rt-p40 1 h before the microbiological stimulus), and LPS+rt-p40 (rt-p40 1 h post-stimulus). Data were obtained from the measurement of 3 independent experiments in triplicate (mean ± SEM).

Next, the cell viability of HaCaT cells was determined after the addition of the LPS microbiological stimulus with and without rt-p40 treatment. However, no statistically significant differences were found between any of the groups studied, as seen in Figure 2.

**Figure 3:**
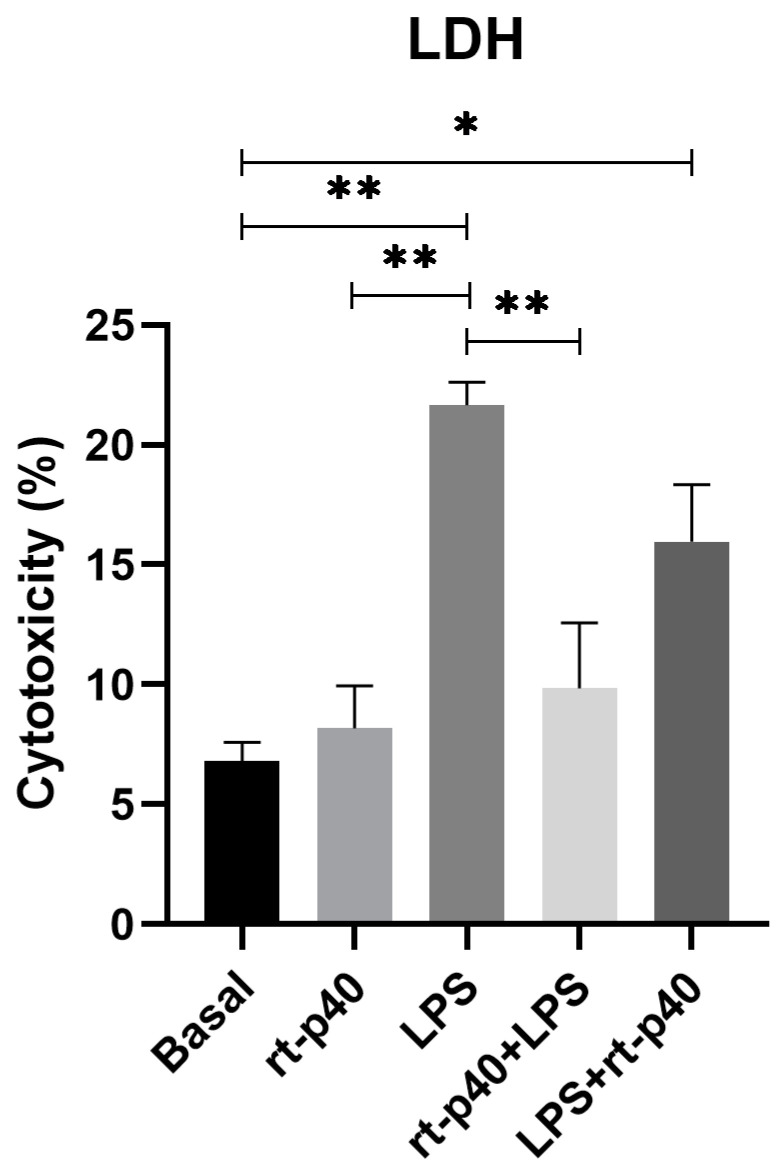
Cell cytotoxicity in the HaCaT cell line under the different stimuli with rt-p40 (10 ng/mL, 1 h), LPS (1 µg/mL, 24 h), rt-p40+LPS (rt-p40 1 h before the microbiological stimulus), and LPS+rt-p40 (rt-p40 1 h post-stimulus). Data were obtained from the measurement of 3 independent experiments in triplicate (mean ± SEM). * *p* < 0.05, ** *p* < 0.01 ANOVA with Tukey’s method for multiple comparisons.

In contrast, results from LDH assays demonstrate that the addition of LPS significantly increases cell cytotoxicity (*p* = 0.0017) compared to basal conditions and rt-p40 (*p* = 0.0035), while rt-p40 does not induce cell death when added to HaCaT cells (*p* = 0.9841). We also found that the addition of rt-p40 prior to LPS reduces the percentage of cell cytotoxicity induced by LPS (*p* = 0.0087), whereas the LPS+rt-p40 group shows significantly increased cytotoxicity in comparison to the basal group (*p* = 0.0401) (Figure 3).

### 3.3. TEER

Following the cell viability experiment, we evaluated the effect of the rt-p40 in the TEER value as a measurement of the epithelial barrier integrity formed by the HaCaT cell line. Measurements were taken at three different sites of the inserts, and the mean value was obtained, out of which the TEER from a blank without cells was subtracted.

These results show that the LPS stimulus was not sufficient to reduce the TEER value significantly compared to the basal group (*p* = 0.9997). Regarding rt-p40, when administered alone, it significantly increases the TEER value of HaCaT cells when compared with the basal group (*p* = 0.0357) and with the group with LPS (*p* = 0.0321).

**Figure 4:**
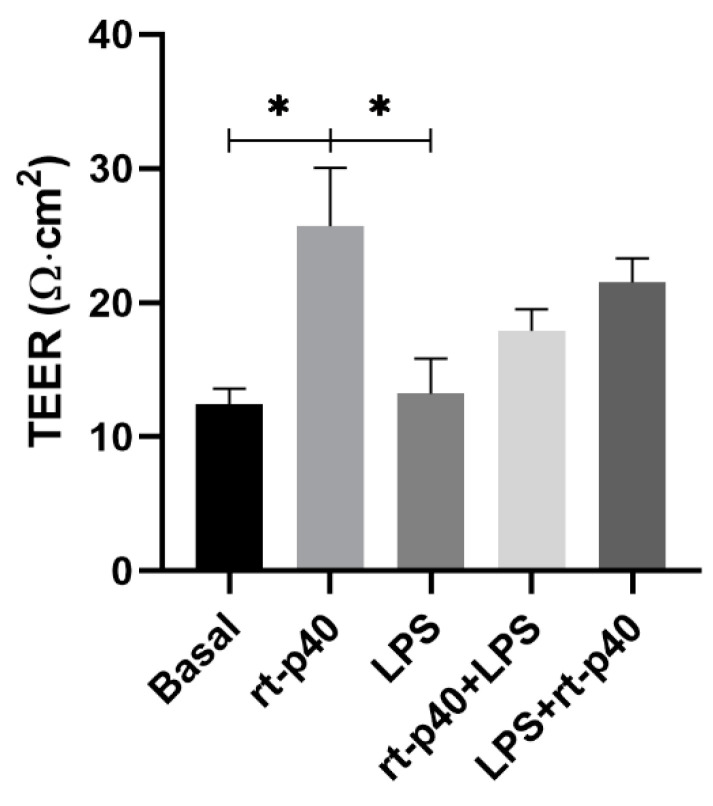
Evaluation of transepithelial electrical resistance (TEER) in the HaCaT cell line. HaCaT cells were cultured for 7 days, and TEER values were obtained under basal conditions, with rt-p40 (10 ng/mL, 1 h), LPS (1 µg/mL, 24 h), rt-p40+LPS (rt-p40 1 h before the microbiological stimulus), and LPS+rt-p40 (rt-p40 1 h after the stimulus). Data were obtained by measuring 3 different sites in each well in 4 independent experiments (mean ± SEM). * *p* < 0.05, ANOVA with Tukey’s method for multiple comparisons.

On the other hand, the combination of rt-p40 with LPS, either before or after this stimulus, did not show statistical significance, even if a slight tendency to increase the TEER value was observed when it was added after LPS (Figure 4).

### 3.4. Expression of Tight Junction Proteins and EGFR Activation

We then analyzed the expression of the tight junction proteins occludin and claudin-1 extracted from the stimulated HaCaT keratinocytes.

**Figure 5:**
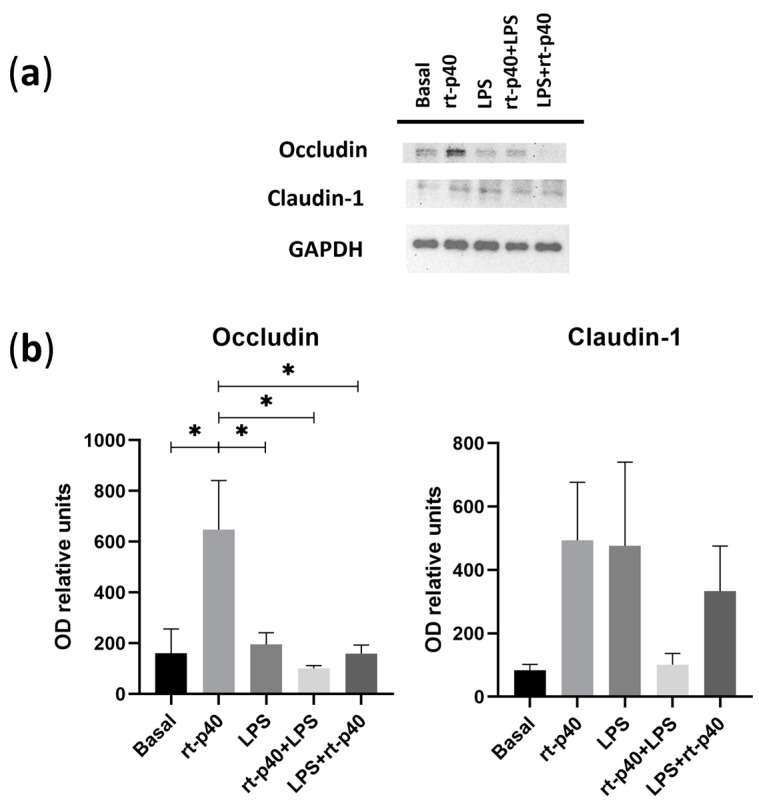
Expression of tight junction proteins in the HaCaT cell line under microbiological stimulation. (**a**) Western blot of occludin and claudin-1 under basal conditions, with rt-p40 (10 ng/mL, 1 h), LPS (1 µg/mL, 24 h), rt-p40+LPS (rt-p40 1 h before microbiological stimulus), and LPS+rt-p40 (rt-p40 1 h after the stimulus). GAPDH was used as a constitutive protein. (**b**) Quantification of the intensity of the occludin and claudin-1 bands, normalized with the band intensity of GAPDH. Data were obtained from 4 independent experiments (mean ± SEM). * *p* < 0.05, ANOVA with Tukey’s method for multiple comparisons.

It was again found that the concentration of LPS was not sufficient to produce a statistically significant change in the protein level of occludin and claudin-1. However, rt-p40 by itself significantly increases occludin expression compared to the basal group (*p* = 0.0247), the LPS group (*p* = 0.0396), the rt-p40+LPS group (*p* = 0.0109), and LPS+rt-p40 (*p* = 0.0245), as observed in Figure 5A. This effect is not observed when rt-p40 is used in combination with the microbiological stimulus. Statistically significant differences in claudin-1 expression were not observed under any of the conditions (Figure 5B).

**Figure 6:**
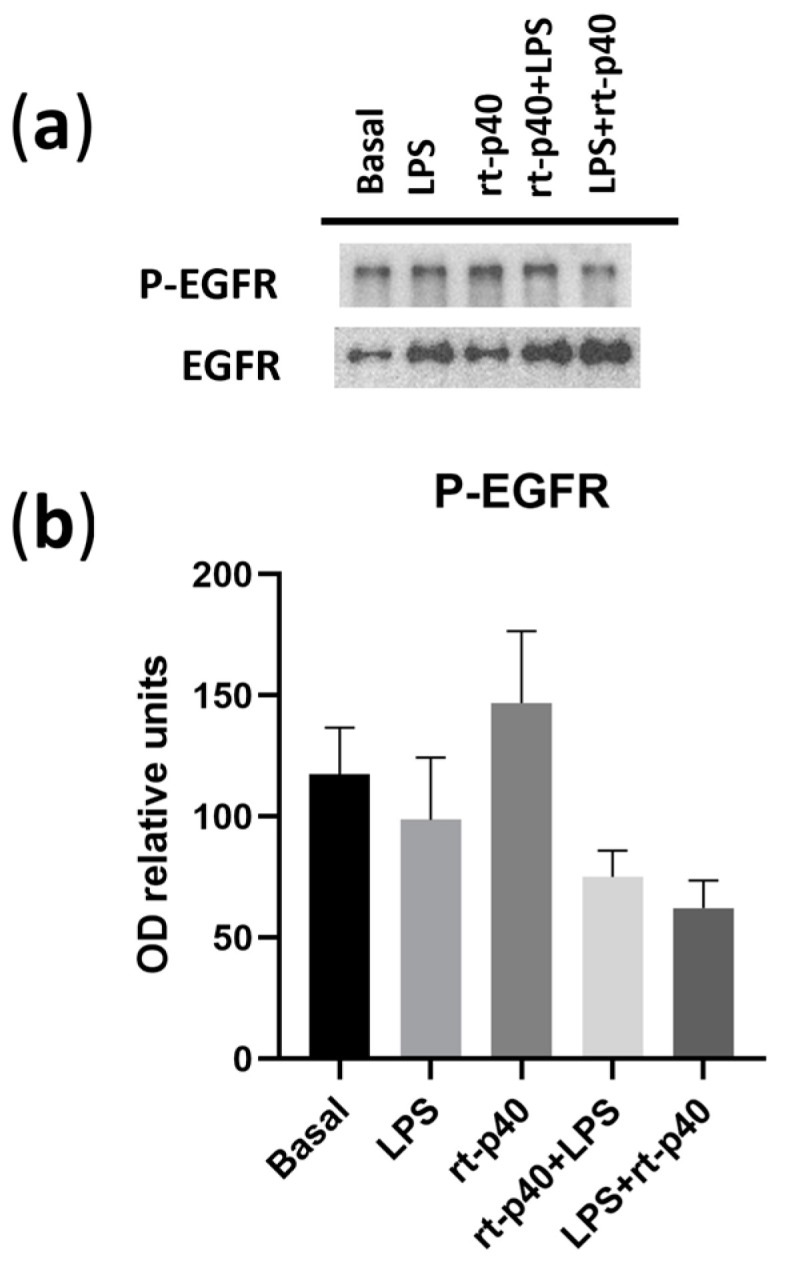
Activation of EGFR in the HaCaT cell line under microbiological stimulation. (**a**) Western blot of P-EGFR (phosphorylated in Tyr 1068 of EGFR) and total EGFR under basal conditions, with LPS (1 µg/mL, 24 h), rt-p40 (10 ng/mL, 1 h), rt-p40+LPS (rt-p40 1 h before the microbiological stimulus), and LPS+rt-p40 (rt-p40 1 h after the stimulus). The band intensity of total EGFR was used to normalize P-EGFR expression. (**b**) Quantification of P-EGFR intensity, normalized to total EGFR band intensity. Data were obtained from 4 independent experiments (mean ± SEM).

We also did not find any statistically significant differences in the activation of EGFR under any of the conditions in our HaCaT keratinocytes (Figure 6).

## 4. Discussion

Postbiotics, molecules derived from bacteria and probiotics, have acquired greater relevance in recent years due to the beneficial effects provided to the health of the host. These effects include anti-inflammatory, immunomodulating, antiproliferative, antioxidant, hypocholesterolemic, antihypertensive, anti-obesogenic, and antimicrobial properties. Therefore, postbiotics have become a more efficient alternative to the use of probiotic bacteria as a treatment, in particular for immunocompromised people [12,13,14,15,16,17,18,19].

Yan et al. reported that the supernatant broth from LGG has anti-apoptotic properties and increases the colonic epithelial cells’ survival in vitro [20]. This effect was due to the two most abundant soluble proteins found in the supernatant. These proteins, p40 and p75, were later purified and proved to be responsible for the anti-apoptotic and anti-inflammatory effects, providing an alternative to the use of probiotics [5]. In particular, the p40 protein presents a remarked improvement in anti-apoptotic, anti-inflammatory, immunomodulatory, and protective effects on intestinal epithelium and in immune cells [5,6,7,21,22,23].

In this work, we achieved the cloning, expression, and purification of rt-p40 from *L. rhamnosus* GR-1, with a change in the 368 amino acids from serine to aspartic acid, in comparison with the reported amino acid sequences of the protein obtained from LGG [5]. This amino acid change is found in the CHAP domain where, according to the three-dimensional structure of the protein, no changes are expected in its structure. Therefore, we expect that its function should not be altered either [24]. Furthermore, rt-p40 is expressed as the mature protein, that is, without the signal sequence for its secretion, which makes it similar to the protein secreted by the bacteria [9,10,25].

The beneficial activity of the p40 protein also involves a protective role for the intestinal epithelial barrier [5,6]. This potential postbiotic protects the intestinal epithelial cells and their tight junctions when subjected to oxidative stimuli, such as H_2_O_2_, maintaining the protein expression of occludin and ZO-1 [6]. Other types of inflammatory stimuli, such as pro-inflammatory cytokines, LPS, and other bacterial metabolites, similarly affect the epithelial barriers of the human body. LPS increases the epithelial barrier permeability in vitro using epithelial cells, where it decreases the TEER value and reduces the expression of the tight junction proteins occludin, claudin-1, and ZO-1 [26,27,28,29].

In our study, we first evaluated the effect of rt-p40 on HaCaT cell viability. We assessed the rt-p40 concentrations of 10, 20, and 50 ng/mL in keratinocytes and performed the MTT assay, with which we found no statistically significant differences in cell viability. This suggests that at these concentrations, rt-p40 does not reduce cell viability in keratinocytes and, thus, could be safe to use. In addition, with this assay, we evaluated the effect of our study conditions, where we found that there were no statistically significant differences in cell viability. Concerning the inflammatory stimulus LPS, a higher concentration could be needed for a statistically significant effect on HaCaT cell viability [30].

Conversely, we determined the effect of LPS on HaCaT cell cytotoxicity, where we found that at concentrations of 1 µg/mL and 10 µg/mL, it significantly increases cell death. Moreover, our results demonstrate that rt-p40 does not induce cell cytotoxicity, and it reduces the LPS-induced increase in cell death when added before the inflammatory stimulus. This is consistent with the study by Yan et al., where p40 from LGG was shown to activate cell survival pathways in colonic epithelial cells [5]. Our rt-p40 protein could also activate these pathways on epidermic epithelial cells, so its use before LPS reduces cell damage induced by this stimulus.

Regarding our results in both MTT and LDH, we noticed a non-significant decrease in viability with LPS in comparison with our basal group, as well as a slight non-significant tendency to increase this viability when rt-p40 was added before and after LPS. These results are complementary to what we report with LDH, with the statistically significant LPS-induced increase in cytotoxicity as well as the reduction in cell death in the rt-p40+LPS group. The apparent differences in both experiments could be due to the basis of MTT, which is a metabolic assay where the activity of dehydrogenase enzymes is measured. While MTT provides an indirect measure of cell activity and viability, it is more accurate to report cytotoxicity effects with other types of assays, such as LDH.

We also measured transepithelial electrical resistance in the HaCaT cell line in four independent experiments. We found no differences from the reported values in the literature, where it is mentioned that HaCaT cells present discontinuous tight junctions and cannot form a paracellular barrier [31,32]. This could explain why our TEER values did not exceed 30 Ω∙cm^2^ and are lower compared to other cell lines, such as the CaCo-2 cell line. On the other hand, we found that the administration of rt-p40 in the HaCaT cell line significantly increases the TEER value compared to the basal group and the group with LPS. This is similar to the reports in CaCo-2 cells with H_2_O_2_ and p40 by Seth et al., where they also found an improvement in TEER values in cells with p40 when administered in the apical and basal cell surfaces [6].

We also found that our rt-p40 significantly increases the protein expression of occludin in HaCaT keratinocytes when administered alone. This also coincides with the findings reported by Seth et al., where p40 protects epithelial barriers. However, unlike the antioxidant effect provided by p40 against H_2_O_2_, the administration of rt-p40 in combination with LPS did not protect or reverse damage [6]. Furthermore, unlike what was reported previously [26,27,28,29], we did not find a statistically significant decrease in the expression of occludin and claudin-1 in this cell line when stimulated with LPS, which could be due to differences in the bacteria strains from which it was obtained.

It is important to highlight that we also noticed an increased protein level of occludin in HaCaT cells after the addition of rt-p40 in comparison with their normal growth conditions. Since the TEER value was also increased, rt-p40 may provide additional stimuli for barrier formation even in HaCaT cells, which have been reported as unable to form an effective paracellular barrier [31,32]. Occludin plays an important role in tight junctions, where the lack of expression of this protein increases paracellular permeability [33], so rt-p40 protein could improve barrier function due to the increase in occludin expression. This also indicates its possible use outside the intestinal barrier for its application in other epithelial barriers that are subjected to inflammatory stimuli.

It has been reported that this p40 transactivates the EGFR pathway in in vitro models with the human colonic cell lines HT-29, T84, and LS174T and with mouse intestinal epithelial cells, as well as in vivo murine models of colitis induced by DSS [7,22,34]. The activation of this receptor is fundamental to preventing pro-inflammatory cytokine-induced apoptosis, decreasing the intestinal epithelial barrier damage induced by oxidative stimuli, and reverting injuries induced by colitis in vivo models. However, we observed that our rt-p40 protein did not activate EGFR when administered to the keratinocyte HaCaT cell line. This could be caused by the differences between the epithelial types from which these cell lines are derived (colonic and epidermal). It is worth mentioning that another possibility is that the colonocytes used in the previously mentioned studies are derived from colorectal adenocarcinomas, while the HaCaT cells are immortalized keratinocytes from healthy tissue.

EGFR is overexpressed in 25–82% of colorectal cancer cases [35]. Therefore, it has been marked as an important molecular target for the treatment of malignant neoplasms since its pathway activates cell proliferation, angiogenesis, migration, survival, and adhesion, all of them critical factors for cancer development [36]. This suggests that the ability of p40 to transactivate this receptor could result in a poor prognosis for some cases since it may be a protective factor for this type of cancer cells.

As prospects, it is necessary to analyze the possible negative effect of this potential postbiotic, p40, in the protection of cancer cells. Furthermore, higher concentrations of rt-p40 may be required to observe notable effects on the protection of tight junction proteins. It is also notable that the signaling pathway in skin epithelial cells seems to differ from the pathway reported for intestinal epithelial cells, thus highlighting the need to research other possible mechanisms of action.

Postbiotics such as p40 represent an attractive alternative for the treatment of various intestinal pathologies, but it is important to check that there are no negative effects before their use in human patients. Likewise, it is notable that the use of this potential postbiotic has only been investigated in the intestinal epithelial barrier. However, our rt-p40 also seems to act positively in other epithelial barriers, such as the skin. Thus, it is important to consider the possibility of using rt-p40 for pathologies that involve the epidermal barrier.

## 5. Conclusions

Postbiotics are soluble factors derived from microorganisms, including probiotics, which represent a possibility of obtaining their benefits without the bacterial-associated risks in the host, especially for people with a vulnerable and compromised immune system [4]. The postbiotic p40 obtained from *L. rhamnosus* GG displays anti-apoptotic and anti-inflammatory properties reported in in vitro and in vivo models with intestinal epithelial cells [5]. In this work, the cloning, expression, and purification of rt-p40 from *L. rhamnosus* GR-1 was achieved, which was then used in HaCaT keratinocytes to evaluate its effect on the LPS-stimulated epidermic epithelial barriers.

By evaluating its effect in this in vitro model, we report that rt-p40 reduces LPS-induced cytotoxicity and increases the value of TEER and the expression of occludin through an EGFR-independent pathway in HaCaT keratinocytes. This particular finding could be due to the different nature of the cell lines, with previous works on the effects of this protein being reported on colonic epithelial cells instead of keratinocytes. Moreover, it is possible that the origin of these cell lines, derived from colorectal adenocarcinomas, could also play a significant role in the activation of this pathway.

Although it is still a recent field, postbiotics have shown great potential for therapeutic applications, out of which p40 is one of the most promising. This work represents the first report of rt-p40 on other types of epithelial barriers, opening the way for future studies of this protein in other cell models. With these findings, it is possible to use this potential postbiotic in pathologies that affect the epidermal barrier, such as atopic dermatitis, skin wound healing, and other epithelial pathologies outside the gastrointestinal tract, like in the respiratory and genitourinary tracts.

## Figures and Tables

**Table 1:** List of primers used for cloning rt-p40.

Name	Sequence	Length (bp)	%GC	TM Q5 °C	Purpose	Reference
P40NCOI	GGGTGAGGTCAATCATGAAATTCAATAAA-GCAATGATGAC	33	48	76	Cloning	This work
P40XHOI	TAAACTCGAGCCGGTG-GATGTAAACGTAGCTG	32	50	74	Cloning	This work

## Data Availability

The data presented in this study are available on request from the corresponding author.

## Supplementary Materials

The following supporting information can be downloaded at: https://www.mdpi.com/article/10.3390/microorganisms11122913/s1. Figure S1: Amino acid sequence alignment of the sequences of our rt-p40, p40 from *L. rhamnosus* GR1, and p40 from LGG; Figure S2: Amino acid sequence alignment between our rt-p40 with p40 from LGG, comparing the differences between them according to the physicochemical properties of the amino acids; Figure S3: Three-dimensional structure of our rt-p40, obtained by the PHYRE2 algorithm; Figure S4: Different concentrations of rt-p40 do not reduce the cell viability of HaCaT keratinocytes. 1 × 10^5^ HaCaT cells were seeded and incubated with 10, 20, and 50 ng/mL rt-p40 for 1 hour. Viability was assessed through the conversion of water-soluble MTT to water-insoluble formazan by viable cells. The formazan crystals were solubilized with DMSO to generate a solution whose color is proportional to the concentration. A negative control without cells was used. Data were obtained from the measurement of 3 independent experiments in triplicate (mean ± SEM); Figure S5: HaCaT cells were stimulated with 0.1–10 µg/mL LPS to determine cell cytotoxicity. A total of 1 × 10^4^ HaCaT cells were seeded and incubated with LPS for 24 hours. Cell cytotoxicity was assessed through LDH release upon damage to the cell membrane. LDH catalyzes the reaction of lactate to pyruvate via NAD+ reduction. NADH is then oxidized by diaphorase to reduce tetrazolium salt to formazan. Formazan is directly proportional to LDH released. Data were obtained from the measurement of 3 independent experiments in triplicate (mean ± SEM).
