# Supplementary material for: Recombinant p40 Protein Promotes Expression of Occludin in HaCaT Keratinocytes: A Brief Communication
Source: Microorganisms. 2023 Dec 3;11(12):2913. doi: 10.3390/microorganisms11122913 (PMC10745755; doi:10.3390/microorganisms11122913)
Supplement: Supplementary file 1 [file microorganisms-11-02913-s001.zip › microorganisms-2714346-supplementary.pdf]

Supplementary material

Amino acid sequence of the recombinant p40 protein (rt-p40) from *L. rhamnosus* GR1

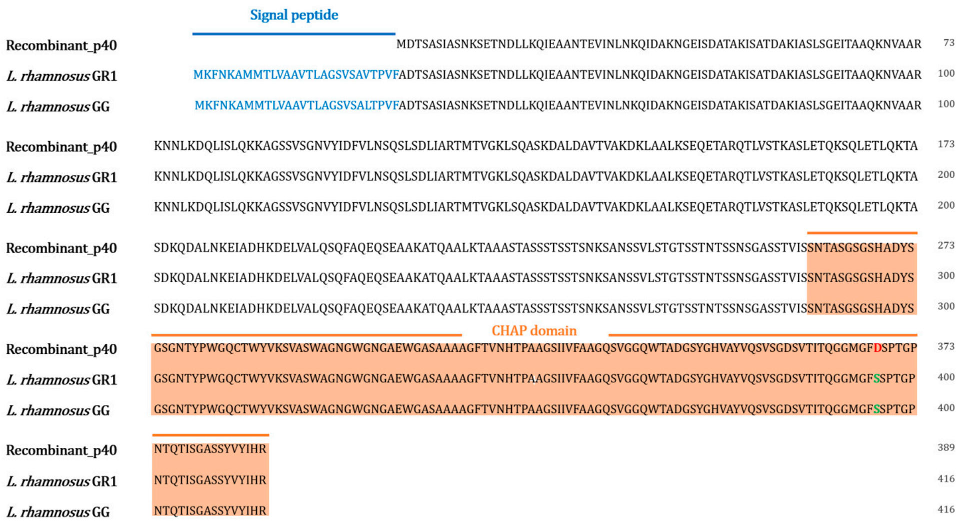

Figure S1. Amino acid sequence alignment of the sequences of our rt-p40, p40 from *L. rhamnosus* GR1 and p40 from LGG.

Comparison of the amino acid sequences between our recombinant p40 protein and p40 from LGG.

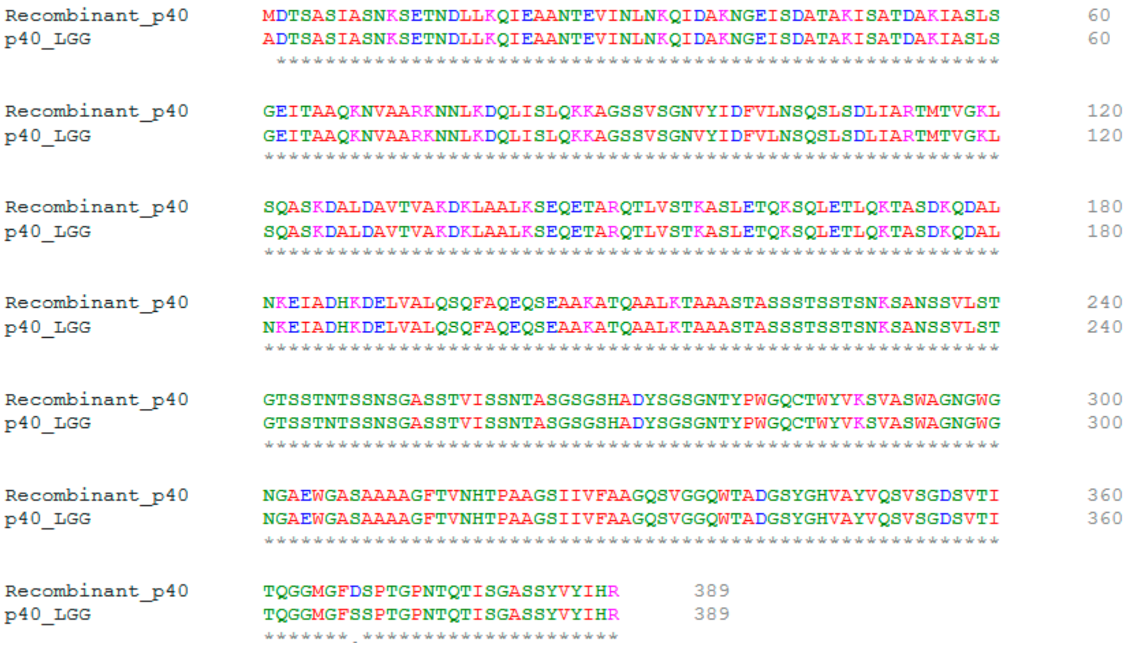

Figure S2. Amino acid sequence alignment between our rt-p40 with p40 from LGG, comparing the differences between them according to the physicochemical properties of the amino acids.

Three-dimensional structure of our rt-p40

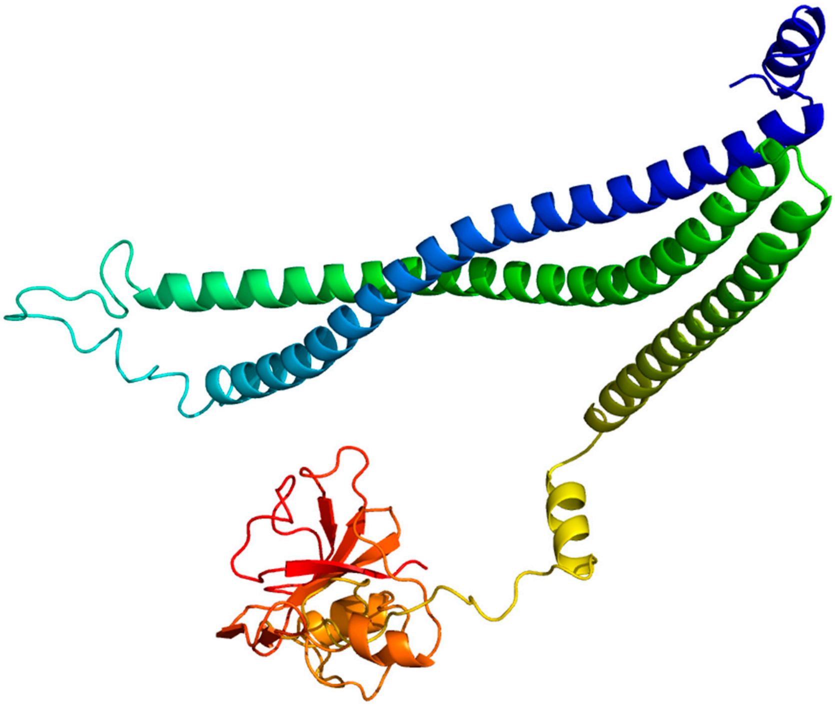

Figure S3. Three-dimensional structure of our rt-p40, obtained by the PHYRE2 algorithm.

Viability assay using the CyQUANT™ MTT Cell Proliferation Assay kit.

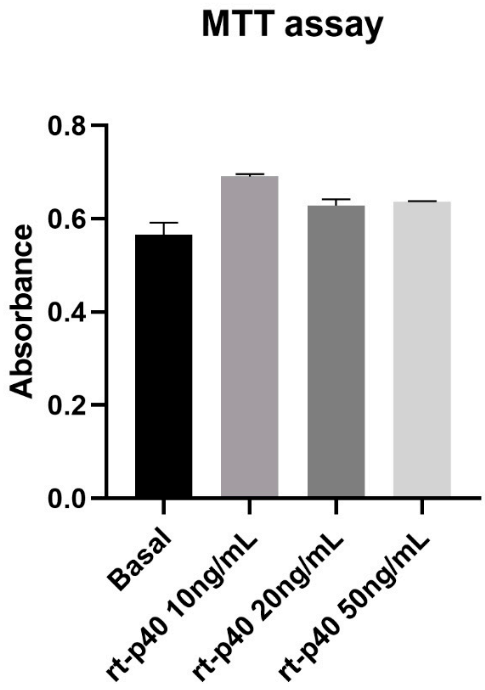

Figure S4. Different concentrations of rt-p40 do not reduce the cell viability of HaCaT keratinocytes. 1×10^5 HaCaT cells were seeded and incubated with 10, 20 and 50 ng/mL rt-p40 for 1 hour. Viability was assessed through the conversion of water-soluble MTT to water-insoluble formazan by viable cells. The formazan crystals were solubilized with DMSO to generate a solution whose color is proportional to the concentration. A negative control without cells was used. Data were obtained from the measurement of 3 independent experiments in triplicate (mean ± SEM).

Cytotoxicity assay using CyQUANT™ LDH Cytotoxicity Assay Kit

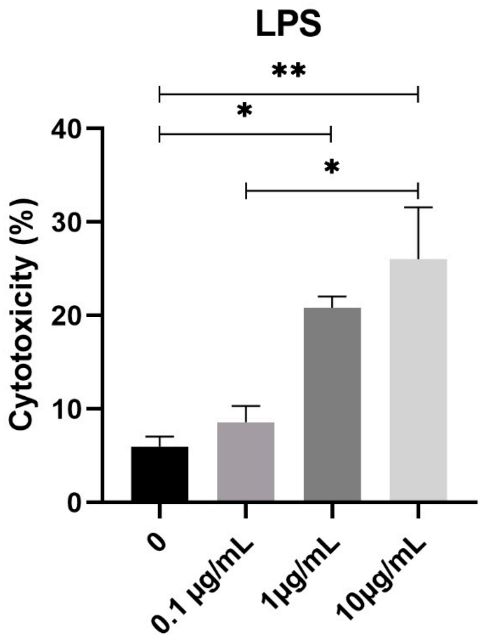

Figure S5. HaCaT cells were stimulated with 0.1 – 10 µg/mL LPS to determine cell cytotoxicity. 1 × 10^4 HaCaT cells were seeded and incubated with LPS for 24 hours. Cell cytotoxicity was assessed through LDH release upon damage to the cell membrane. LDH catalyzes the reaction of lactate to pyruvate via NAD+ reduction. NADH is then oxidized by diaphorase to reduce tetrazolium salt to formazan. Formazan is directly proportional to LDH released. Data were obtained from the measurement of 3 independent experiments in triplicate (mean ± SEM).
